# Factors associated with compliance to recommended micronutrients uptake for prevention of anemia during pregnancy in urban, peri-urban, and rural communities in Southeast Nigeria

**DOI:** 10.1186/s41043-016-0068-7

**Published:** 2016-11-02

**Authors:** Nkechi G. Onyeneho, Ngozi I’Aronu, Ngozi Chukwu, Uju Patricia Agbawodikeizu, Malgorzata Chalupowski, S. V. Subramanian

**Affiliations:** 1 Department of Global Health and Population, Takemi Program in International Health, Harvard TH Chan School of Public Health, 677 Huntington Avenue, Boston, MA USA; 2 Department of Sociology/Anthropology, University of Nigeria, Nsukka, Enugu State Nigeria; 3 Department of Social Work, University of Nigeria, Nsukka, Enugu State Nigeria; 4 Harvard School of Public Health, 677 Huntington Avenue, Boston, MA USA; 5 Department of Social and Behavioral Sciences, Harvard School of Public Health, 677 Huntington Avenue, Boston, MA USA

**Keywords:** Anemia, Pregnancy, Compliance, Micronutrients

## Abstract

**Background:**

The study investigated the factors associated with compliance to the recommended ≥90-day uptake of micronutrients for prevention of iron-deficiency anemia during pregnancy in Nigeria.

**Methods:**

A cross-sectional study of 1500 women who had babies within 6 months prior to the survey, drawn from six urban, peri-urban, and rural local government areas in Enugu and Imo States of Nigeria, was conducted, using a structured questionnaire. A focus group discussion was held with grandmothers and fathers of the new baby. In-depth interviews were held with health workers.

**Results:**

There were six demographic factors in the bivariate analysis: living in an urban center and close to health facility, and being wealthy, with post-secondary education as well as older and engaged in civil service showed significant association with compliance. The urban residents complied more than the peri-urban and rural residents (*χ* ^2^ = 12.749; *p* = 0.002). Those living close to the health facilities complied more than those living far away (*χ* ^2^ = 24.638; *p* < 0.001). Those in higher wealth quintile complied more (*χ* ^2^ = 13.216; *p* < 0.010). Utilization of antenatal clinics during pregnancy showed statistically significant association with compliance. Those who used the ANC services complied more than those that did not (*χ* ^2^ = 6.324; *p* = 0.010) and the more frequent the use of ANC services the more the compliance (*χ* ^2^ = 14.771; *p* < 0.001). These results were confirmed when the opinions expressed in the urban, peri-urban, and rural communities are compared. However, the multivariate binary logistic regression highlighted only urban residence, closeness to health facilities, and utilization of ANC services as positively associated with compliance.

**Conclusion:**

These findings could help in targeting health education program to increase compliance to the recommended uptake of micronutrients in prevention of anemia during pregnancy.

## Background

Maternal mortality ratio (MMR) remains high in sub Saharan Africa and indeed Nigeria despite substantial reduction global trends in maternal deaths [1–3]. In Nigeria, for instance, MMR increased from 545 to 575 deaths per 100,000 live births between 2008 and 2013 reflecting a worsening situation and failure in achieving the fifth target of the MDG [4–6]. Anemia has been implicated as a major driver of MMR in Nigeria and other developing countries [7]. WHO pegs the values at 10.0–10.9 g/dl (mild anemia), 7–9.9 g/dl (moderate anemia), and <7 g/dl (severe anemia) [8, 9].

Anemia in pregnancy (AIP) is one of the most serious global public health problems[10, 11]. WHO estimates that more than half of pregnant women globally have a hemoglobin level indicative of anemia (<11.0 g/dl) [10]. Fifty-two percent of pregnant women in developing countries compared with 23 % in the developed world have anemia in pregnancy [10]. AIP prevalence varies considerably because of the differences in socio-economic conditions, lifestyles, and health-seeking behaviors across different cultures [11].

Prevalence of anemia in Nigeria is put at more than 40 % [11] among pre-school age children, pregnant women, and non-pregnant women of reproductive age alike. It is thus classified as severe and an important public health problem in Nigeria. In a hospital study in Southeastern Nigeria, Dim and Onah [12] reported that 40.4 % of the pregnant women registered with the antenatal unit were anemic (hemoglobin [Hb] <11.0 g/dl). Okafor and Rizzuto had earlier warned that hospital estimates of any pregnancy-related problem are often an under-estimation of the true prevalence given that most women deliver outside the health facilities in Nigeria [13].

Iron deficiency, the most prevalent cause of anemia, often coexists with deficiency in other important micronutrients, a link which makes it more deadly [14–20]. Nutritional deficiencies such as anemia are often worsened by the additional nutrient demands associated with fetal growth. Inclusion of iron supplements in foods can improve diets, control intestinal parasites, and protect women against iron-deficiency anemia during pregnancy. Unfortunately, only 21 % of women took iron tablets daily for 90 or more days during their last pregnancy in Nigeria [4].

Writing in the Guardian newspaper, Ezeonjiaku quoted the Nutrition Society of Nigeria (NSN) as blaming iron deficiency for high prevalence of anemia among Nigerian women. According to the NSN, the country faces one of the largest burdens of micronutrient deficiencies in the world, with anemia and its health impact the most common, where approximately 50 % of cases of anemia in Nigeria are due to iron deficiency. The society further argued that adolescent girls and pregnant women are the populations requiring the highest amount of iron intake and are, therefore, most susceptible to iron deficiency [21]. Forty-nine percent of women of reproductive age have anemia, 24.3 % have low iron stores, and 2.7 % of them are iron deficient. Majority of girls and women do not meet the WHO and FAO recommended iron requirements of 20 mg per day. Pregnant women, teenage girls, and women of reproductive age are highly vulnerable to iron-deficiency anemia because of high iron requirements [21].

“In Nigeria, mothers are the kitchen ‘gatekeepers’ and their adolescent daughters learn cooking behaviors from them,” (Samuel cited in Ezeonyejiaku, [21]). According to Samuel, many Nigerian women are aware of iron-rich sources of food in the environment, its benefits, and consequences of iron deficiencies, yet the consumption of iron-rich food is still low. A typical Nigerian diet is low in iron-rich foods, but high in phytates, which decreases iron absorption.

Compliance studies on other medical conditions reveal that adhering to a medical regimen may be influenced by the characteristics of the patient/client and provider as well as the nature of the regimen and may guide thinking about compliance with recommended uptake of food supplements to prevent iron-deficiency anemia during pregnancy [22–24]. Some of the factors associated with compliance with recommended regimen in preventing other medical conditions include patient factors such as ethnic origin, educational attainment, as well as knowledge and perceptions about the conditions and solutions [22–27]. However, a systematic review of published randomized and quasi-randomized trials in PubMed and Cochrane library revealed paucity of literature on factors associated with micronutrients uptake, especially in the developing countries [28].

This study presents results on an examination of factors associated with levels of compliance with uptake of iron supplements to prevent anemia among pregnant women with varying socio-demographic and cognitive characteristics. The conclusion of the study would help in designing programs to ensure compliance with recommended micronutrient intake during pregnancy to prevent iron-deficiency anemia during pregnancy.

### Understanding micronutrient supplementation: evidence from literature

**Table:** 

*Search strategy and selection criteria*
We searched google scholar and the WHO Global Information Full Text (GIFT) system using the search terms “anemia”, “anemia”, and in combination with “in pregnancy”, “during pregnancy”. Other terms like “compliance”, “deficiency”, and “micronutrient” were also used. We focused on all publications, including reviews and publications in their draft forms. We also searched reports from WHO, World, and UNICEF. We reviewed reference lists of publications and reports identified by this search strategy and selected those that addressed micronutrients and their uptake during pregnancy published between 2000 and 2015 and to which we had access to the full paper either in pdf or doc formats.

High prevalence of anemia among pregnant women is reported in Africa despite the availability of effective interventions to prevent anemia during pregnancy. A number of studies put the prevalence of anemia in pregnancy within Africa at between 57 and 86 % [10–12, 19, 29–35]. This is blamed on both nutritional deficiency and infections. An editorial in 2001 noted that nutritional deficiency contributes 32 %. A study in eight rural districts of Ethiopia revealed that women lacked comprehensive knowledge of anemia [36].

Given the widespread prevalence of micronutrient deficiencies in developing countries, supplementation with multiple micronutrients rather than iron-folate alone could be of potential benefit to the mother and the fetus. Haider et al. evaluated evidence from 17 articles on impact of multiple micronutrient supplements during pregnancy compared with standard iron-folate supplements on specific maternal and pregnancy outcomes of relevance to the Lives Saved Tool (LiST) [37, 38]. The results show that there is no significant difference between the multiple supplementation and iron supplement alone.

Yakoob et al. conducted a systematic review of published randomized and quasi-randomized trials on PubMed and the Cochrane Library as per the CHERG guidelines [28]. They concluded that there is a paucity of studies of intermittent iron of iron-folate supplementation, especially in developing countries. They thus recommended further evaluation of this intervention in comparison with daily supplementation regimes.

A cross-section study in Mali, to assess pregnant women’s acceptability of and adherence to a daily multiple micronutrient supplementation scheme compared with the current daily iron and folic acid supplementation scheme, revealed that women started receiving supplements at the end of the first trimester of pregnancy until delivery. Adherence to the multiple micronutrient supplementation scheme was better than that to the iron and folic acid supplementation scheme. However, both were very good, as were women’s perceptions about the benefits of micronutrient supplements to their health and that of their newborns [39].

Ogundipe et al. assessed the factors associated with prenatal folic acid and iron supplementation among 21,889 pregnant women in Northern Tanzania, employing cross-sectional hospital-based data [40]. They observed that folic acid and iron supplementation were low among the women in Northern Tanzania. They identified certain factors as positively associated with folic acid supplementation to include advanced maternal age, unknown HIV status, and being diagnosed positive of anemia.

Miller et al. examined the impact of agricultural practices on micronutrients in the food supply, including cropping systems, soil fertility, and animal agriculture [41]. They also explored successful strategies to preserve micronutrients from farm to plate, including food fortification. The exercise ended with recommendations for actions that will reduce the prevalence of micronutrient malnutrition.

Darnton-Hill et al. conducted a review of literature to identify the micronutrients (vitamins and minerals) likely to be deficient in women of reproductive age in low- and middle-income countries (LMIC), especially during pregnancy, and the impact of such deficiencies [42]. In a theoretical paper on micronutrient in pregnancy, Black enumerated the many consequences of micronutrient deficiency in pregnancy and recommended additional research on the prevalence of such deficiencies and their consequences and on cost-effective public health interventions for their control [43].

In a cross-sectional study, Shipala et al. assessed the adequacy of nutrient intake including proteins, energy, calcium, iron, folate, and vitamin C and identified the factors associated with nutrient intake in Kenya [44]. The results revealed that intake of all selected nutrients were significantly lower than the required dietary allowance. Protein intake was significantly associated with education, income, and perceived food shortage. Energy intake was significantly associated with income. Iron intake was significantly associated with perceived food shortage. Similar studies by Gomez et al. in a cohort of Alberta pregnant women and Horan et al. in their ROLO study and Christian et al. in rural Nepal highlight the need for both dietary and supplemental sources of micronutrients when assessing the nutrient intakes of pregnant women [45–47].

The common feature of all the studies is the heavy dependence on statistical approach with limited qualitative data that provides the rich context of the behavior of the women vis-à-vis compliance with the recommended micronutrient uptake during pregnancy. This present study employed the mixed method approach in collection of data from women in different environments, namely rural, peri-urban, and urban settings. The adoption of the mixed-method approach provided opportunity for the exploitation of the rich synergy in triangulating information from qualitative and quantitative methods of inquiry.

## Methods

### Study design

The study was designed for description and analysis of the knowledge levels, attitudes, and practices concerning anemia during pregnancy, using mixed methods in urban, peri-urban, and rural communities. The study combined two analytical designs, namely (1) a cross-sectional survey and (2) qualitative inquiry. The survey research was based on an interviewer-administered household survey instrument while the qualitative inquiry was based on in-depth interviews and focus group discussion methods. These two designs contribute to an in-depth, triangulated understanding of community knowledge, attitudes, and practices on anemia during pregnancy in Southeast Nigeria.

### Study settings, population, and sampling

**Fig. 1:**
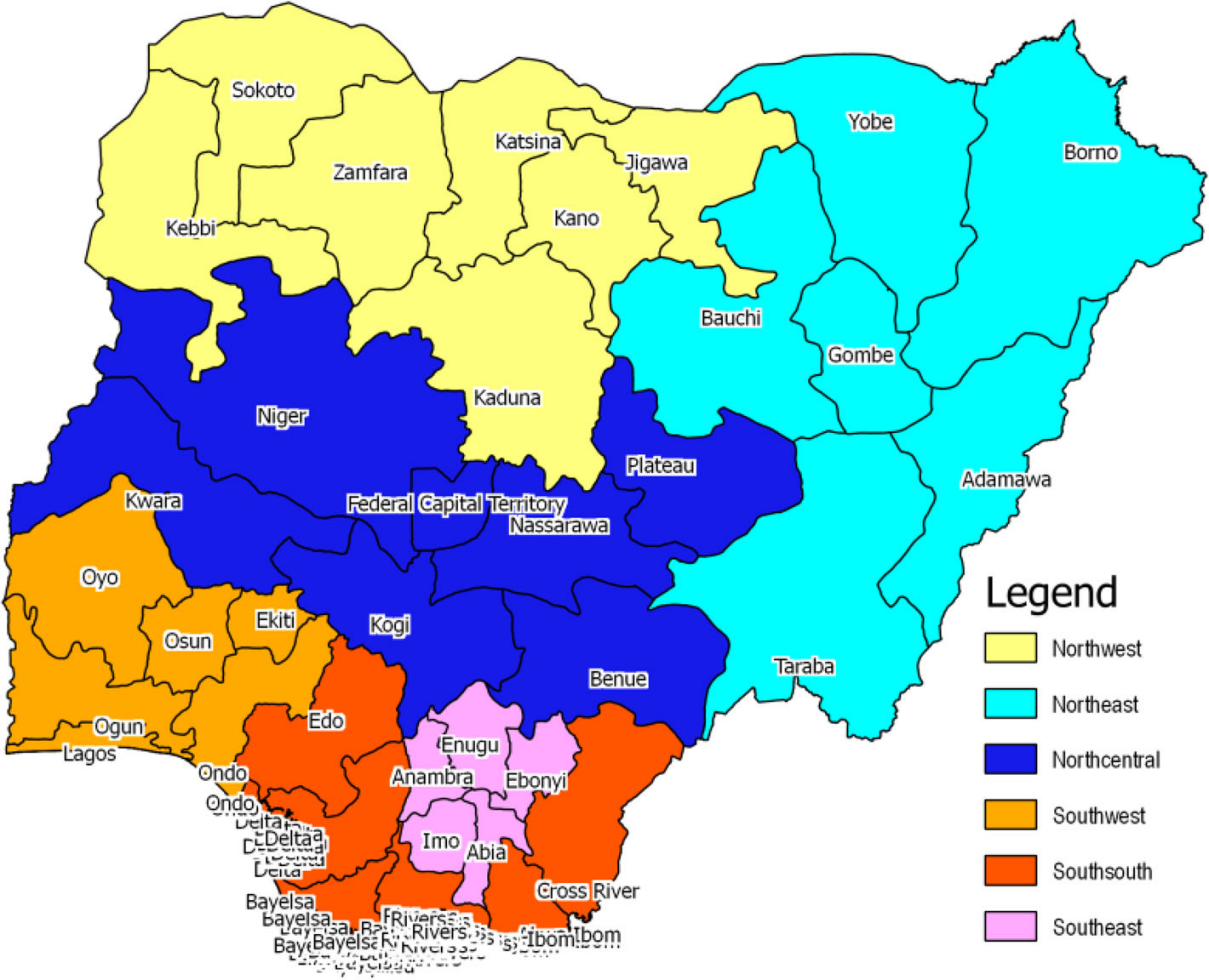
Map of Nigeria showing the geopolitical zones and the study sites

The Nigerian political structure comprises of 36 states and the Federal Capital Territory distributed into six geopolitical zones (GPZs). See Fig. 1. These GPZs are made up of states with semi-autonomous health systems that independently develop strategies for attaining health targets, in line with the national health policy. The GPZs represent broad clusters of political, cultural, and linguistic realities in Nigeria. They are the fundamental context for understanding Nigeria’s ethnic diversity. The realities, represented by this spread, go a long way to influence the health services and behavior of the people. The South East GPZ (comprising of five states) is predominantly Igbo, one of the three largest ethnic-sociocultural groups in Nigeria. Beyond being culturally homogenous, the zone is well known to the researcher. Beyond being culturally homogenous, the zone had the highest average zonal immunization coverage on all basic vaccines for children (51.7 %) in 2013, although the value is below the desired 80 % expected at the local government area (LGA) levels. The other zones—South West, South-South, North Central, North East, and North West—had average zonal coverage of 40.9, 52.0, 26.9, 14.2, and 9.2 %, respectively [4].

States in the South East GPZ were grouped into two categories of “strong” (well performing) versus “weak” (less well performing) based on immunization coverage with all basic vaccines for children, as a reflection of the PHC development at the state and LGA levels, which has implication for health awareness and behavior of mothers during pregnancy. The zonal average all basic vaccination coverage for the South East was 51.7 %. Thus, any state within the zone with less than the zonal average was classified as weak. Imo State, with 62.4 is classified as “strong,” while Abia (49.8 %), Anambra (51.6 %), Ebonyi (51.1 %), and Enugu (45.0 %) were classified as “weak.” Imo is purposively selected to represent the strong health system while Enugu State was randomly selected from cluster of weak performing states.

Enugu State is located between latitude 6°30′ N and longitude 7° 30′ E. It is a mainland state in southeastern Nigeria (http://en.wikipedia.org/wiki/Enugu_State). Its capital is Enugu in Enugu North LGA. Carved out of the old Anambra State in 1991, the principal cities in the state are Enugu, Udi, Oji, and Nsukka. The state shares borders with Abia and Imo States to the South, Ebonyi State to the East, Benue State to the Northeast, Kogi State to the northwest, and Anambra State to the West. The 2006 population census put Enugu State population at 3,257,298 [48]. With an annual growth rate of 2.35 %, the 2015 population of Enugu State was put at 4,014,654 persons. Approximately 50 % (50.1 %) is made up of women, out of which 43.9 % fall within the child bearing age of 15–49 years.

University of Nigeria Teaching Hospital (UNTH) is located in Enugu State, as is the Enugu State University Teaching Hospital and College of Medicine. In addition to numerous private hospitals and clinics in the state, there are seven district hospitals in Enugu Urban, Udi, Agbani, Awgu, Ikem, Enugu-Ezike, and Nsukka. There are 46 government-owned health facilities located in seven health districts.

Imo State lies between latitude 4°45′ and 6°15′ N and longitude 6°30′ and 8°09′ E and is bounded by Abia and Anambra States on the North. Rivers and Bayelsa States bound it in the South. Imo State has an estimated total population of about 4,849,311 million persons in 2015 based of 2006 population figure of 3,934,499, with 27 local government areas. Imo State is culturally distinct area with a remarkable health seeking behavior. There are a number of health institutions that provide primary, secondary, and tertiary health care in the state.

The primary unit of analysis for this study is all mothers of child bearing age (15–49 years). It focused in understanding the practices to prevent anemia during pregnancy. It is noted that iron status can be enhanced by including iron supplements in foods consumed by women, improving women’s diets, and controlling intestinal parasites. In Nigeria, only 21 % of women take iron tablets daily for 90 or more days during their last pregnancy. In Enugu and Imo States, only 64.7 and 47.8 % respectively did so. The southeast zonal intake of iron tablets for 90 or more days was 38.3 % [4].

The LGAs in each of the two selected states will be classified and grouped into three clusters of rural, urban, and peri-urban. Three LGAs, one each from the urban, peri-urban, and rural segments of each sampled state, were randomly selected. From the list of communities in each sampled LGA, two communities were randomly selected. These communities formed the sampling clusters from which eligible respondents, who delivered within 6 months preceding the survey, were drawn, following a two-stage sampling. The communities were grouped into two clusters of far (>5 km) and near (<5 km) to the LGA PHC center. One community was randomly selected from each cluster, yielding a total of 12 communities.

Using a 40.4 % rate of occurrence of anemia in pregnancy in southeastern Nigeria [12] and a confidence interval of 95 % with an estimated 2.5 % level of precision, a sample size of 1480 ± 37 respondents was computed in the communities. However, the sample size was rounded up to 1500 households, taking into account a 1 % contingency rate. Approximately 125 women were interviewed in each of the 12 communities.

To select the households, a central location in each of the randomly selected communities was identified to serve as the starting point for data collection in the selected community. Two data collectors were assigned to cover each community cluster and the interviewers moved in opposite directions from the identified starting point in each community. Interviewers continued to turn right at every junction, until the desired number of respondents is attained.

### Research instruments

A structured interview schedule (other-administered questionnaire) constituted the main instrument for data collection. It covered information on the socio-demographic characteristics of the respondents as well as their child bearing experiences. It also provided data on the women’s experiences with the PHC as well as their knowledge, attitude, and practice concerning anemia during pregnancy among others. The questionnaire consisted mainly of close-ended questions with lists of optional responses from which the respondents chose their responses. However, given the focus of the study to ascertain the knowledge, attitude, and practices of the respondents on anemia, they were not allowed prior knowledge of the content of the questionnaires; hence, the other-administered approach was used. The respondents gave their answers as they considered best. Where the answers they gave did not fall within the options, the responses were classified under “others.”

Some qualitative data were collected with the use of focus group discussion (FGD) and in-depth interview (IDI) guides. The FGD and IDI explored people’s opinion, perceptions, and attitudes toward anemia during pregnancy. They contained open-ended guide questions with probes providing wider exploration of the opinions, perceptions, and attitudes expressed by respondents to IDIs and participants in FGD sessions.

The FGDs were health with the husbands and mothers of the women aged 15–49 years, who delivered babies within 6 months preceding the study. There were a total of six FGDs for husband and six FGDs for mothers of the women who delivered within 6 months preceding the study. Each FGD session consisted of 8 to 12 participants. These were the primary support and significant others to the women who delivered within 6 months preceding the study. They are privileged to know practices of the women during their pregnancy and delivery. It was not possible to get sufficient number of the women, who delivered within 6 months preceding the study, to a convenient place for FGD. Thus, their husbands and mothers served as proxy.

On the other hand, IDI were held with persons in charge of the health facilities in the communities. A total of six IDI sessions were held with the health workers. Being the providers of health care, especially ANC services in the communities, they possessed knowledge of the actual practices and attitudes toward anemia in pregnancy among women of child bearing ages in the communities.

### Statistical analysis

Data management and analysis were performed using EPI Info version 6.04d and the statistical package for social sciences (SPSS) version 22. Statistical significance was assessed using two-tailed tests. All *p* values less than 0.05 were considered to be statistically significant. Knowledge scores were assigned to each participants corresponding with the number of correct answers on knowledge questions. The numeric scores were tabulated for various groups and then re-categorized as binary variables, either greater than or less than the mean knowledge score. Similar actions were taken to derive both the attitude and practice scores.

Categorical variables were presented as frequencies and percentages. Chi-square (*χ* ^2^) tests were used to assess the statistical association between variables. Mean scores of anemia in pregnancy knowledge, attitudes, and practices were compared among socioeconomically different communities as well as population with varying demographics. Binary logistic regression analyses were used to adjust for known covariates. Bi-variate association analyses were used to select the candidate-associated factors for the multivariate logistic regression. The multi-categorical variables were converted to dummy variables to calculate odds ratio of each value. The principal component analysis (pca) models were employed to compute the wealth index.

## Results

Table 1 presents a summary of the key socio-demographic characteristics of the respondents to the questionnaire (*N* = 1500). Equal numbers (500 respondents) came from the urban, peri-urban, and rural backgrounds, respectively. These were also equally distributed across six local government areas (LGA) from Enugu and Imo States, in Nigeria. The respondents were also equally shared between those residing far from, and near to the community health facilities. They were mainly Igbo (98.0 %).

**Table 1:** Distribution of respondents by socio-demographic information and locality (% are in brackets)

Socio-demographic information	Locality			
	Urban	Pre-urban	Rural	Total
Local gov’t areas				
Ezeagu	–	–	250 (50.0)	250 (16.7)
Ihite Uboma	–	250 (50.0)	–	250 (16.7)
Nsukka	250 (50.0)	–	–	250 (16.7)
Obowo	–	–	250 (50.00)	250 (16.7)
Owerri Municipal	250 (50.0)	–	–	250 (16.70)
Udi	–	250 (50.0)	–	250 (16.7)
Distance from community health facility				
Far	250 (50.0)	250 (50.0)	250 (50.0)	250 (50.0)
Near	250 (50.00)	250 (50.0)	250 (50.0)	250 (50.0)
Tribe				
Igbo	489 (97.8)	487 (97.4)	494 (98.8)	1470 (98.0)
Others	11 (2.2)	13 (2.6)	6 (1.2)	30 (2.0)
Religious affiliation				
Catholic	307 (61.4)	272 (54.4)	334 (66.8)	913 (60.9)
Protestant	109 (21.8)	105(21.0)	91 (18.2)	305 (20.3)
Other Christian	82 (16.4)	118 (23.6)	75 (15.0)	275 (18.3)
Islam	2 (0.4)	4 (0.8)		6 (0.4)
Tradition		1 (0.2)		1 (0.1)
Marital status				
Married	491 (98.2)	496 (99.2)	485 (97.0)	1472 (98.1)
Widowed	4 (0.8)	0 (0.0)	2 (0.4)	6 (0.4)
Divorced	0 (0.0)	0 (0.0)	2 (0.4)	2 (0.1)
Single	5 (1.0)	4 (0.8)	11 (2.2)	20 (1.3)
Ever attended school				
Yes	496 (99.2)	497 (99.4)	494 (98.8)	1487 (99.1)
No	4 (0.8)	3 (0.6)	6 (1.2)	13 (0.9)
Level of education	*n* = 497	*n* = 496	*n* = 494	*N* = 1487
Primary	26 (5.2)	32 (6.5)	60 (12.1)	118 (7.9)
Secondary	227 (45.7)	345 (69.6)	368 (74.5)	940 (63.2)
Post secondary	244 (49.1)	119 (24.0)	66 (13.4)	429 (28.9)
Work for pay	*n* = 500	*n* = 500	*n* = 500	*N* = 1500
Yes	355 (71.0)	373 (74.6)	364 (72.8)	1092 (72.8)
No	145 (29.0)	127 (25.4)	136 (27.2)	408 (27.2)
Primary occupation	*n* = 355	*n* = 373	*n* = 364	*N* = 1092
Domestic assistance	3 (0.8)	3 (0.8)	3 (0.8)	9 (0.8)
Petty trading	87 (24.5)	104 (27.9)	157 (43.1)	348 (31.9)
Business	154 (43.4)	199 (53.4)	157 (43.1)	510 (46.7)
Teaching	40 (11.3)	23 (6.2)	24 (6.6)	87 (8.0)
Health worker	21 (5.9)	17 (4.6)	14 (3.8)	52 (4.8)
Other civil service	33 (9.3)	22 (5.9)	7 (1.9)	62 (5.7)
Other	17 (4.8)	5 (1.3)	2 (0.5)	24 (2.2)
Age at last birthday	*n* = 500	*n* = 500	*n* = 500	*N* = 1500
Mean	*29.01*	*27.97*	*28.42*	*28.47*
STD	*4.97*	*5.31*	*5.71*	*5.35*
15–19	12 (2.4)	15 (3.0)	27 (5.4)	54 (3.6)
20–24	72 (14.4)	115(23.0)	94 (18.8)	281 (18.7)
25–29	183 (36.6)	190 (38.0)	169 (33.8)	542 (36.1)
30–34	164 (32.8)	118 (23.6)	132 (26.4)	414 (27.6)
35–39	55 (11.0)	49 (9.8)	59 (11.8)	163 (10.9)
40–44	12 (2.4)	11 (2.2)	17(3.4)	40 (2.7)
45+	2 (0.4)	2 (0.4)	2 (0.4)	6 (0.4)

Christianity of all types dominated the sample with only 6 (0.4 %) and 1 (0.1 %) Islam and traditionalists respectively, sampled. Over 90 percent (98.1 %) of the respondents were married. Almost every person interviewed had one form of formal education or another (99.1 %). However, almost half (49.1 %) of the urban respondents had post-secondary education while smaller proportions (24.0 and 13.4 %) of the peri-urban and rural respondents respectively had post-secondary education.

Most (72.8 %) of the people interviewed were engaged in income generating activities, mainly in the form of business (46.7 %) and petty trading (31.9 %), followed by teaching (8.0 %) and other civil service (5.7 %). The distribution cut across different localities of urban, peri-urban, and rural. However, slightly more (74.6 and 72.8 %) of the respondents from the peri-urban and rural backgrounds respectively than their urban counterparts (71.0 %) indicated that they were engaged in paid employments The mean age of the respondents in the entire sample was 28.47 years (28.47 ± 5.35SD). The age distributions were fairly uniform across the urban, peri-urban, and rural samples.

Principal component analysis was used to compute an asset index and used to divide the respondents’ households into five asset quintiles. This was based on possession of items such as refrigerator, motor cycle, motor car, and radio. The asset index was adapted from the Nigeria Demographic and Health Survey. It contained 11 household items, namely whether the household has access to electricity, landline telephone, refrigerator, radio, and television. Other items included cell (smart) phone, bicycle, motorcycle, motor car, tractor, and livestock. There was a normal distribution around the second quintile with 35.5 % of the respondents. More (24.8 %) of the urban dwellers than their peri-urban (7.8 %) and rural counterparts (4.6 %) were found the richest (fifth) wealth quintiles, respectively. Conversely, more of the rural dwellers (32.2 %) than their peri-urban and urban (10.4 %) counterparts fell within the poorest quintile.

**Fig. 2:**
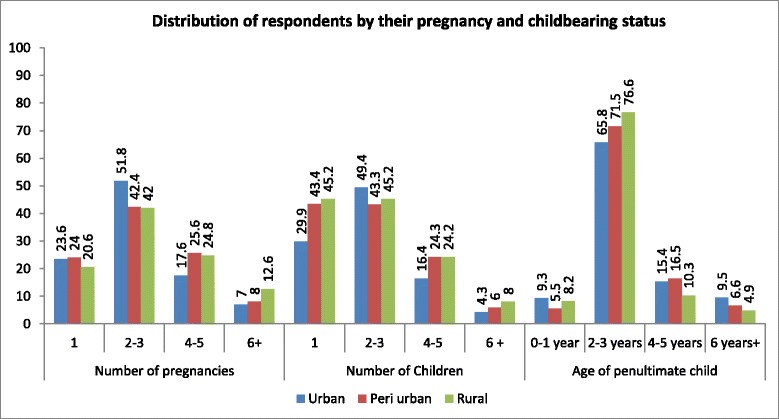
Distribution of respondents by their pregnancy and childbearing status

The child bearing practices were similar across the three localities. The number of pregnancies across the samples ranged from 1 to 10 among the urban and peri-urban respondents, respectively, while it ranged from 1 to 11 with a mean of 3.22 pregnancies (3.22 ± 1.95SD) among the rural respondents. The mean number of pregnancies for the urban respondents was 2.76 children (2.76 ± 1.68SD). The mean number of pregnancies among the peri-urban respondents was 2.90 (2.90 ± 1.67SD) pregnancies. Figure 2 showed that a higher proportion (12.6 %) of the rural respondents have had more than five pregnancies, compared to the peri-urban (8.0 %) and urban (7.0 %).

**Fig. 3:**
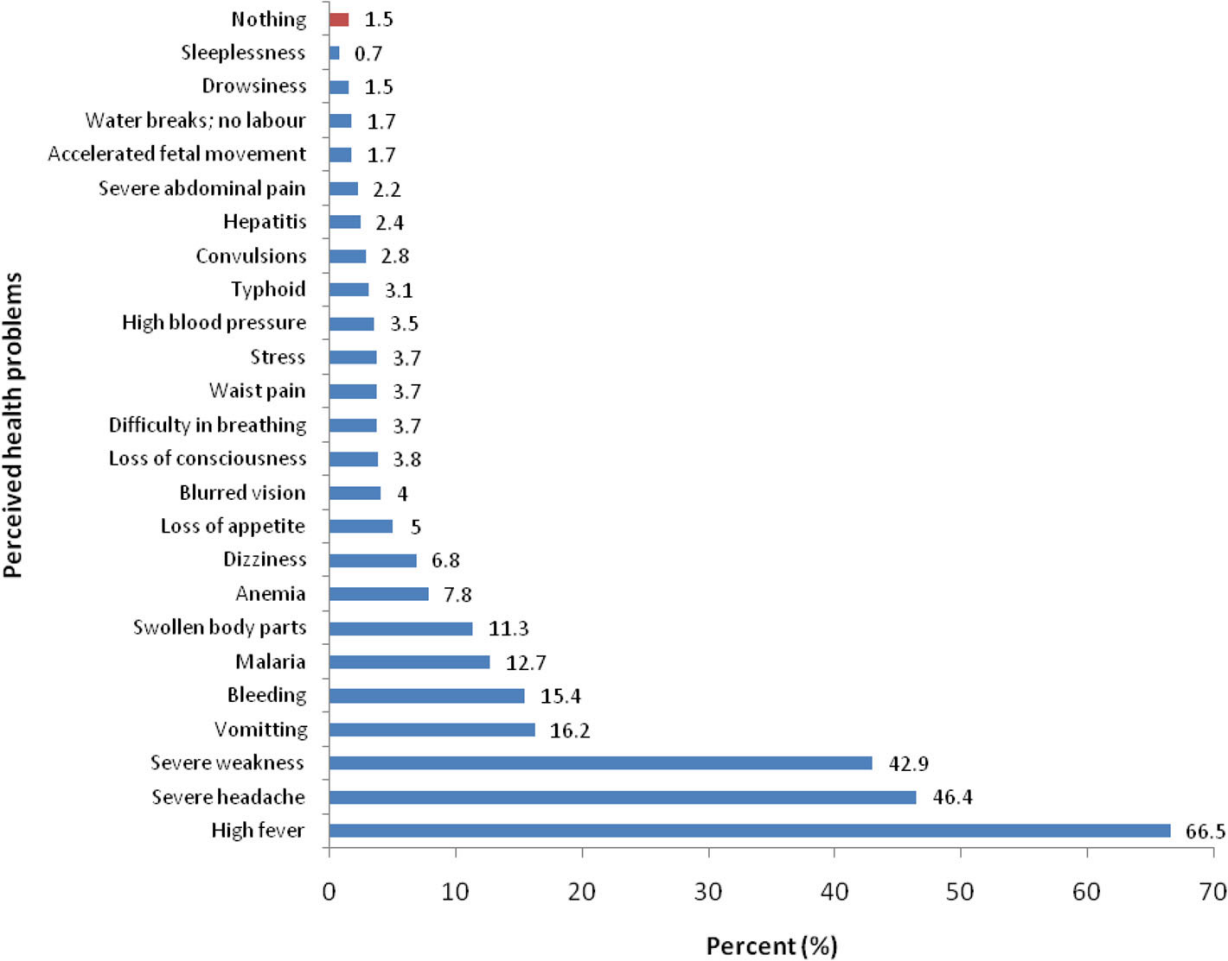
Distribution of respondents by perceived health problems

**Fig. 4:**
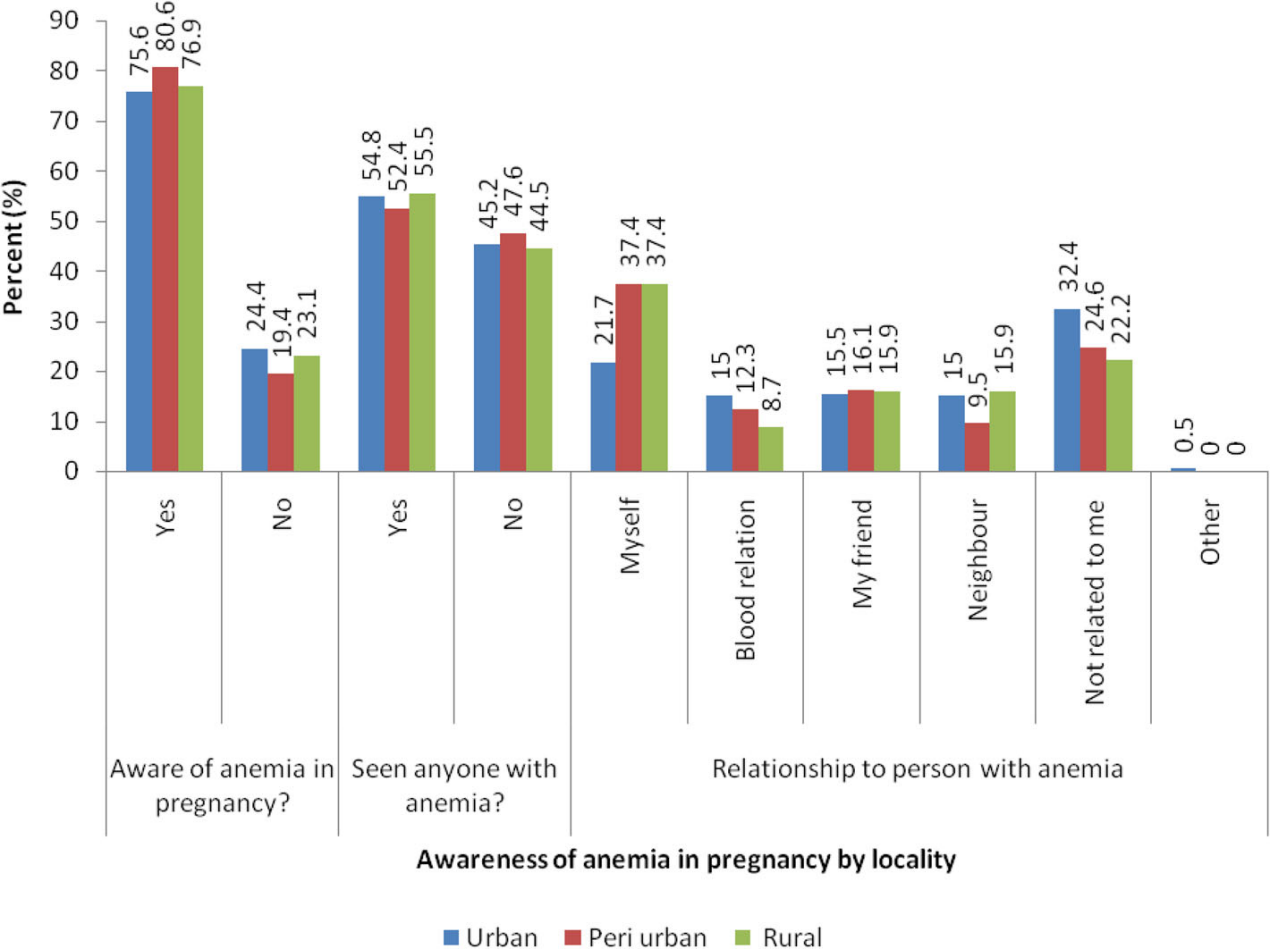
Distribution of respondents by awareness of anemia in pregnancy by locality

To an unprompted question, only 7.8 % of the respondents indicated awareness of anemia as a problem among women of reproductive ages, while almost all the respondents indicated malaria and fever (Fig. 3). However, when asked specifically on their awareness of anemia in pregnancy, 75.5 % of the urban respondents indicated awareness of the anemia while 80.6 and 76.9 % of the peri-urban and rural respondents respectively indicated awareness of anemia in pregnancy (Fig. 4).

When prompted with question of whether they are aware of anemia, participants in the qualitative study indicated that they are aware of anemia. However, for them, it is mostly in pregnant women and is seen as normal and nothing to worry about. The following quotes are typical of the feelings of the participants to anemia during pregnancy as a reality that should be lived with and not cause panic.

It is something that happens because a woman is pregnant so once it is said that the blood is not at a good level, the woman will be advised on how to build up the blood again. Although many women are asked to eat vegetables to increase their blood level, it is not really anemia because anemia means that there is very little blood in the woman’s body. In this community it does not get that bad because people are educated and therefore take care of themselves. Again, mostly people who receive salaries live here so they would usually not be too poor to eat well **[FGD: Fathers, Owerri urban]**

Anemia is one of the signs of pregnancy because it shows that the woman’s body is working for two people. It is even better to have anemia in pregnancy than to have other diseases like diabetes high blood pressure or typhoid **[FGD: Grandmothers, Ihitte peri urban]**

About 80 % (79.1 %) of the respondents took micronutrient supplements to prevent anemia during their last pregnancy. More (83.2 %) of the urban residents than the peri-urban (76.0 %) and the rural (78.0 %) took micronutrients to prevent anemia during their last pregnancy. The median number of days they took the micronutrients was 98, 90, and 60 days for the urban, peri-urban, and rural respondents, respectively. Table 2 shows that compliance with the recommended uptake of micronutrients for ≥90 days was more among the urban respondents, where 59.8 % complied (*χ* ^2^ = 12.749; *p* = 0.002).

**Table 2:** Distribution of respondents by selected background variables and compliance with recommended (90 days or more) dose of iron supplements to prevent anemia during pregnancy

Background variables	Categories	Number	% up take for 90 days+	Chi-square
				*p* values
Locality	Urban	336	59.8	12.749
	Peri-urban	328	51.5	0.002
	Rural	287	45.6	
Distance from health facility	Far	454	44.3	24.638
	Near	497	60.4	<0.001
Tribe	Igbo	928	52.7	0.002
	Non-Igbo	23	52.2	0.563
Religious affiliation	Catholic	565	52.4	2.779
	Protestant	204	51.5	0.595
	Pentecostal	177	55.4	
	Islam	4	25	
	Traditionalist	1	100	
Marital status	Married	937	52.9	6.111
	Widowed	2	100	0.106
	Divorced	2	0.0	
	Single	10	30.0	
Level of schooling	Primary	72	41.7	7.600
	Secondary	587	51.4	0.022
	Post-secondary	286	58.4	
Employed	Yes	697	53.5	0.728
	No	254	50.4	0.218
Employment type	Domestic aid	8	12.5	21.002
	Petty trading	204	47.5	0.002
	Business	334	53.0	
	Teaching	52	59.6	
	Health worker	38	55.3	
	Civil servant	43	74.4	
	Other	18	77.8	
Age group	15–19	32	37.5	12.653
	20–24	192	47.4	0.049
	25–29	331	49.8	
	30–24	265	58.5	
	35–39	100	58.0	
	40–44	25	64.0	
	45+	6	66.7	
Number of pregnancies	Once	235	48.9	7.461
	2–3 times	429	51.0	0.059
	4–5 times	198	61.1	
	6 times and above	89	51.7	
Knowledge of anemia in pregnancy	Good (≥9 points)	522	53.6	0.426
	Poor (<9 points)	429	51.5	0.278
Wealth index	First quintile	188	48.4	13.216
	Second quintile	333	50.2	0.010
	Third quintile	178	48.3	
	Fourth quintile	138	63.8	
	Fifth quintile	114	60.5	
Antenatal care visit	Yes	939	53.1	6.324
	No	12	16.7	0.012
Number of ANC visits	<4 times	882	54.4	14.771
	≥4 times	69	30.4	<0.001

Compliance was better among those who lived close to health facilities (60.4 %) than among those who lived far away from health facilities (44.3 %) (*χ* ^2^ = 24.638; *p* < 0.001). Compliance was better among those with post-secondary education (58.4 %) than those with secondary (51.4 %) and primary (41.7 %) education, respectively (*χ* ^2^ = 7.600; *p* = 0.022). The older the age of the women, the better the compliance (*χ* ^2^ = 12.653; *p* = 0.049).

The employment and wealth statuses of the women also influence compliance with ≥90 days uptake of micronutrients during the last pregnancy. Those engaged in civil service (74.4 %) or teaching (59.6 %) complied more (*χ* ^2^ = 21.002; *p* = 0.002). The higher the wealth quintile of the women the more they complied (*χ* ^2^ = 13.216; *p* = 0.010). Number of births had no influence on the women’s compliance with the ≥90-day recommended uptake of micronutrients to prevent anemia during the last pregnancy. Utilization of antenatal care services also influenced compliance.

The qualitative data highlighted some of the food intake and supplementation women use to protect themselves against anemia during pregnancy. This shows some variation by social background namely the education and economic statuses of the women.

The ones that come here are educated on the types of food to eat and we also give the iron tablets which is part of the routine drugs we give each one of them **[IDI: Health worker; UDI peri-urban]**

But those who don’t have money settle for the local means of building the blood. They can use green vegetables or make juice out of bitter leaves. Yes, when they make the juice out of vegetables, they either add malt to it and this helps to build the blood **[FGD: Grandmother; UDI]**

The nurses always tell them to eat vegetable and take the drug they gave at the clinic. But there are lots of things that can be done to quickly replace blood for a pregnant woman. She can eat lots of fresh tomatoes, she can also mix tin tomato puree especially Derica and milk (peak milk), this one works instantly. There are also people who mix the blood of tortoise and milk or you can also add malt drink to it. These things help a pregnant woman to give her blood until she has the baby **[FGD: Grandmothers; Ezeagu rural].**

In the village, where I live, the women will be advised to make juice out of Ugu vegetable (fluted pumpkin). They can also use bitter leaf or moringa leaves to make juice. Ehen they make this juice, they can add malt or milk and these help to build the blood very quickly **[FGD: Grandmothers; Owerri Urban]**

**Fig. 5:**
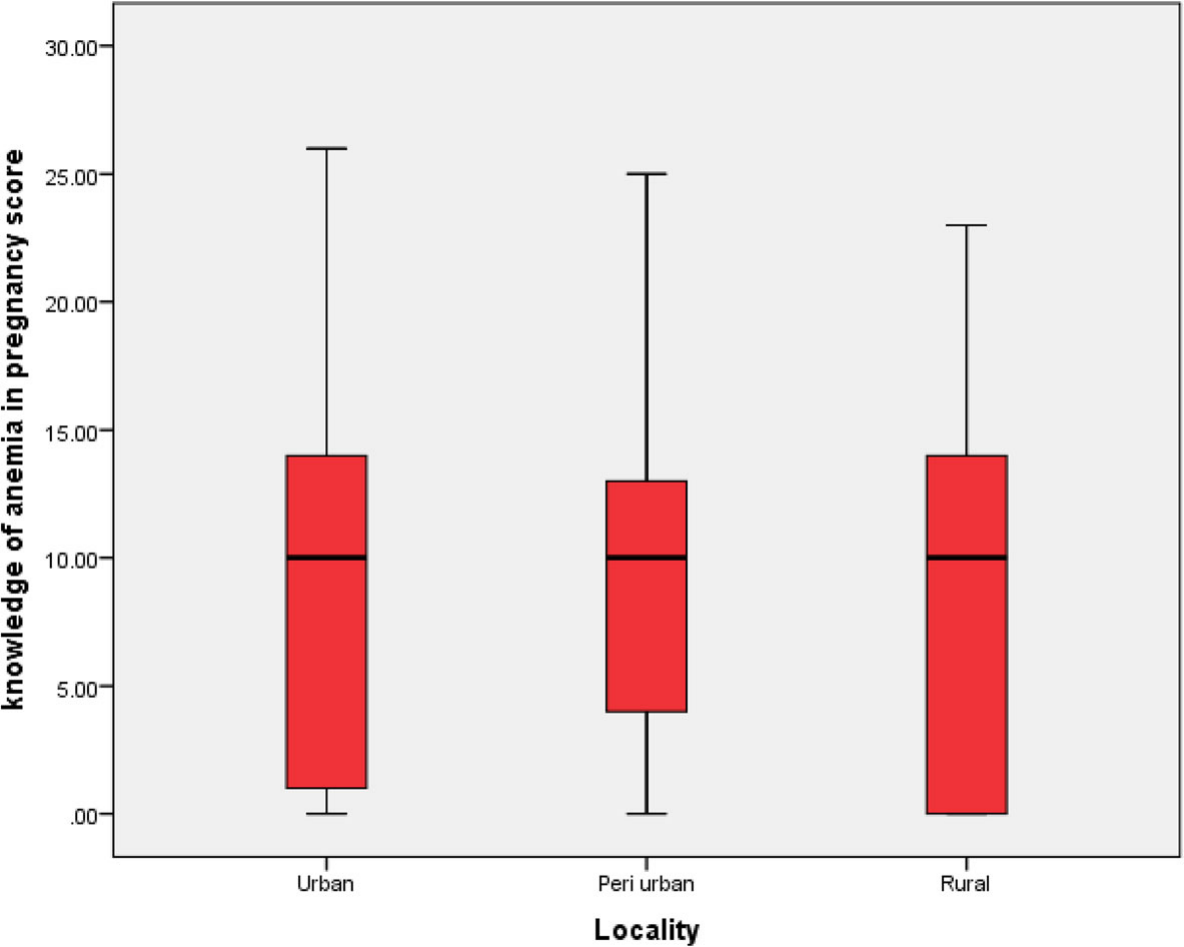
Knowledge score on anemia in pregnancy by locality

**Fig. 6:**
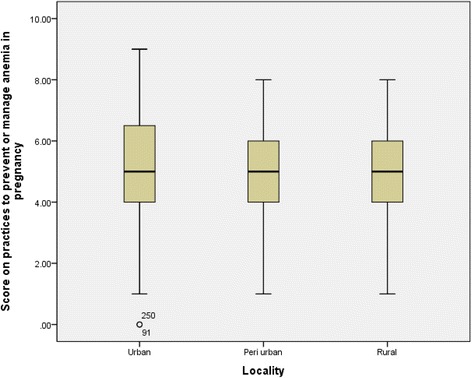
Score on practices to prevent or manage anemia in pregnancy by locality

A knowledge index comprising of 50 variables drawn from perceived causes, how anemia affects pregnancy, ways of preventing anemia, signs of anemia, and management of anemia was constructed Figs. 5 and 6. The scientifically proven responses were entered in the index, as binary variables, where each correct response takes a score of “1” and incorrect response takes a score of “0.” The knowledge score ranged from 0 to 26 points with an average score of 8.866 (8.866 ± 6.3STD). The average score of 8.866 points was chosen for a cutoff of good and poor knowledge. A score of ≥9 points was classified as good taking the binary score of 1, else the respondent is classified as having poor knowledge of anemia in pregnancy taking the binary score of 0. The result showed that 53.6 % of those with good knowledge (≥9 point) compared to 51.5 % of those with poor knowledge (<9 point) complied.

The low knowledge level on anemia in pregnancy also came out clearly in the qualitative study as can be attested to with the following quotes with respect to management:

There are some leaves you can get from the bush. Once you wash them, blend them into juice and drink, it will build the blood. You can also use the normal vegetable we eat in the homes. Just eat a lot of it and you will be ok. They also give them tablets that can give blood **[FGD: Grandmothers, Nsukka urban]**

There are certain things that need to be done. For instance, there is need for sensitization, public education on the dangers of AIP. Then, the women should be given nets to prevent malaria. Again these routine drugs should be supplied to them free of charge **[IDI: Health worker Owerri Urban]**

With respect to protection, a husband in an FGD session with father had this to say:

They are not protected from getting anemia because you can’t avoid it. It is like asking if you can protect them from having big stomach when they are pregnant. You know it is not possible. But the health center gives them drugs and we give them whatever they like to eat. My wife likes to eat biscuit and Pepsi anytime she is pregnant so I buy biscuit and Pepsi for her. I don’t think that gives her shortage of blood because the human body grows well when you eat the things you really like **[FGD: Fathers; Ezeagu Rural]**

Regression analysis identified factors associated with compliance to ≥90-day recommended uptake of micronutrients (Table 3). Two demographic factors, namely living in an urban area and being close to health facility as well as ANC practices, showed positive association with compliance. It showed that a unit increase in the urban experience of the women would lead to 0.296 unit increase in compliance to the recommended uptake of micronutrients during pregnancy. Similar results were observed with closeness.

**Table 3:** Key factors associated with compliance to ≥90-day uptake of micronutrients during pregnancy

Factors		*B*	S.E.	Wald	df	Sig.	Exp(*B*)	95 % C.I. for EXP(*B*)	
								Lower	Upper
	Urban	0.296	0.149	3.915	1	0.048	1.344	1.003	1.801
	Near health facility	0.640	0.135	22.523	1	0.000	1.896	1.456	2.469
	Post-secondary education	0.070	0.165	0.181	1	0.670	1.073	0.777	1.482
	Civil service	0.327	0.204	2.553	1	0.110	1.386	0.929	2.069
	35 years and above	0.220	0.197	1.249	1	0.264	1.246	0.847	1.834
	Third quintile and above	0.144	0.142	1.025	1	0.311	1.155	0.874	1.525
	Attended ANC clinic	1.781	0.790	5.083	1	0.024	5.933	1.262	27.899
	ANC ≥ 4 times	0.894	0.276	10.479	1	0.001	2.446	1.423	4.204
	Constant	−3.085	0.837	13.572	1	0.000	0.046		

## Discussion

Nutritional deficiency is the commonest cause of AIP [10–12, 14–20, 29–35]. Micronutrients accessible from ANC clinics are capable of controlling the situation. However, uptake is low, especially in Nigeria [4, 5]. Unfortunately, clarity is yet to be achieved on the factors the drive uptake of micronutrients and existing literature revealed on anemia in pregnancy reveal paucity of studies on factors associated with low uptake of micronutrients. This study was thus designed to explore the factors linked to compliance with recommended uptake of micronutrients to prevent anemia during pregnancy in Nigeria, using a mixed method approach.

The study showed low uptake of micronutrients during pregnancy despite their availability in ANC clinics, due in part to low use of the public health facilities for focused antenatal care (FANC). While many mothers make at least one ANC visit during pregnancy, very few do at least four visits, which WHO recommended to ensure FANC with complete anemia prevention care [4, 5, 49, 50]. The analysis revealed that less than four fifths of the respondents took micronutrients during the last pregnancy. Worse still, not all that took micronutrients did so for the recommended ≥90 days, and this varied with the social background of the respondents. For instance, respondents with more urban background complied with the recommended uptake and vice versa (Table 4).

**Table 4:** Distribution of respondents by Good knowledge, prevention and management practice on anemia in pregnancy and selected background variables

Background Variables	Categories	No.	% Good knowledge	Chi sq	% Good practice	Chi sq
				*P* values		*P* values
Locality	Urban	500	47.6	0.905	33.7	1.702
	Peri urban	500	47.6	0.636	32.7	0.427
	Rural	500	45.0		34.6	
Distance from health facility	Far	750	44.3	3.666	49.0	0.526
	Near	750	49.2	0.031	51.0	0.500
Tribe	Igbo	1470	47.2	6.734	98.2	0.317
	Non Igbo	30	23.2	0.007	1.8	0.712
Religious affiliation	Catholic	913	45.1	9.208	60.1	10.551
	Protestant	305	53.8	0.056	23.4	0.032
	Pentecostal	275	44.4		16.1	
	Islam	6	33.3		0.3	
	Traditionalist	1	100		0.1	
Marital status	Married	1472	47.1	7.623	98.7	3.570
	Widowed	6	50.0	0.054	0.4	0.312
	Divorced	2	0.0		0.0	
	Single	20	20.0		0.9	
Level of schooling	Primary	118	40.7	18.318	6.7	6.783
	Secondary	940	43.7	<0.001	61.5	0.034
	Post secondary	429	46.9		31.8	
Employed	Yes	1092	529	4.715	73.2	0.112
	No	408	172	0.017	26.8	0.391
Employment type	Domestic aid	9	77.8	20.497	0.8	9.659
	Petty trading	348	48.9	0.002	30.3	0.140
	Business	510	43.3		45.3	
	Teaching	87	57.5		8.7	
	Health worker	52	61.5		4.6	
	Civil servant	62	62.9		7.9	
	Other	24	41.7		2.4	
Age group	15-19	54	33.3	14.203	3.4	4.632
	20-24	281	43.8	0.027	16.9	0.592
	25-29	542	45.2		36.1	
	30-34	414	48.8		29.7	
	35-39	163	50.9		10.9	
	40-44	40	67.5		2.5	
	45+	6	50.0		0.4	
Number of pregnancies	Once	341	44.0	7.214	22.5	2.077
	2-3 times	681	44.6	0.065	43.8	0.556
	4-5 times	340	50.9		24.0	
	6 times &above	138	53.6		9.8	
Wealth index	First quintile	312	17.8	10.070	20.6	1.346
	Second quintile	535	36.1	0.030	35.5	0.854
	Third quintile	263	19.4		16.9	
	Fourth quintile	204	14.8		14.6	
	Fifth quintile	186	11.8		12.4	
Knowledge of anemia in preg.	Good (11+)	701			62.1	117.190
	Poor (0-10)	799			37.9	<0.001

Studies on compliance with public health programs have identified that certain sub-groups have lower compliance based on social and demographic characteristics [26, 31–35, 51–55], and this study confirms the same problem with the prevention of anemia in pregnancy, thereby identifying subgroups of women who could benefit from more focused health education (Table 5). This study revealed positive association between use of ANC services and compliance with recommended uptake of micronutrients. It showed that women who use ANC services complied more. Furthermore, the more they used ANC services, the more they complied. In addition there is the issue of finance. In many cases, pregnant women are expected to pay for the food supplements. This might explain why overall wealth is associated with compliance. Those in the higher wealth quintile complied more. From the qualitative data, we found that those who come to ANC are educated on the need for micronutrients and are given these micronutrients as routine medications and encouraged to comply. There were also statements that suggest that women who lack money limit women’s choice and uptake of the micronutrients. Then of course is the factor of personal choice, which in many cases is linked to lack of information. The more adherence to FANC the more they get enlightened to take informed decisions on AIP and micronutrients. But this adherence to FANC is affected by attitude of HW which drives them to private for profit. Closely linked to this is distance, which may also imply some monetary costs. All of these, paying for services in private for profit outfits and transportation costs, may explain why more of those in higher wealth quintile are seen to comply more. The general value of greater health education was demonstrated and suggests that health education sessions at ANC and through community outreach needs to be strengthened.

**Table 5:** Binary Logistic Regression on Factors associated with knowlegde and practices on anemia in pregnancy

Factors	Knowledge of anemia in pregnancy			Anemia in pregnancy prevention and management practices		
	Univariate OR (95 % CI)	Multivariate logistic regression		Univariate OR (95 % CI)	Multivariate logistic regression	
		B	Sig.		B	Sig
Distance (near)	0.900 (.998-1.206)	.230	.030	--	--	--
Tribe (Igbo)	0.494 (.258-.948)	1.123	.011	--	--	--
Married	0.530 (.279-1.009)	.920	.040	--	--	--
Post secondaryeduc	0.779 (.699-.869)	.373	.005	0.065 (.769-.972)	.146	.222
Religion (Catholic)	--	--	--	1.034 (.923-1.159)	-.012	.911
Knowledge (Good)	--	--	--	0.535 (.475-.602)	1.140	.000
Employed	0.870 (.765-.990)	.154	.223	--	--	--
Civil service	0.729 (.644-.825)	.437	.012	--	--	--
Age (35 years+	0.842 (.733-.968)	.268	.080	--	--	--
Wealth index (3-5)	0.902 (.810-1.005)	.058	.599	--	--	--
Constant	--	-2.595	.000	--	-.780	.000

The results further underscore the importance for ANC attendance whether there are community volunteers in a program or not. Not only is ANC a place where micronutrient education takes place, but also many of the women got their supplies during ANC sessions. The qualitative data revealed health workers indicating that they recommend these food supplements and also issue same to those that attend ANC. ANC also offers an important opportunity for health education on the broader risks of anemia in pregnancy.

## Conclusions

In conclusion, this study has identified important issues, namely distance, urbanization, ANC attendance, maternal age, and personal financial opportunity, associated with compliance with the recommended ≥90-day uptake of micronutrient in prevention of iron-deficiency-related anemia during pregnancy. Further qualitative study could help confirm some of the challenges in terms of the social barriers associated with different age groups and the role of “work type” in women’s decisions and ability to comply with uptake of food supplements in prevention of anemia during pregnancy. With this additional information, health education strategies can be strengthened to close the gaps identified.

## Abbreviations

AIP: Anemia in pregnancy
ANC: Antenatal care
FANC: Focused antenatal care
FAO: Food & Agricultural Organization
FGD: Focus group discussion
GIFT: Global Information Full Text
GPZ: Geopolitical zone
HIV: Human immunodeficiency virus
IDI: In-depth interview
KM: Kilometer
LGA: Local government area
LiST: Lives Saved Tool
LMIC: Low- and medium-income countries
MDG: Millennium Development Goal
MMR: Maternal mortality ratio
NSN: Nutrition Society of Nigeria
PHC: Primary health care
RTF: Rich Text Format
SGA: Small for gestational age
UNICEF: United Nations Children’s Fund
UNTH: University of Nigeria Teaching Hospital
WHO: World Health Organization
